# Surgical management of subhepatic perforated appendicitis: a case report 

**DOI:** 10.1186/s13256-020-02499-2

**Published:** 2020-09-12

**Authors:** Mumin Hakim, Rania Mostafa, Mohammed Al Shehri, Sherif Sharawy

**Affiliations:** Department of General Surgery, Saudi German Hospital, Al-Aseer, Kingdom of Saudi Arabia

**Keywords:** Subhepatic appendicitis, Peritonitis, Laparotomy, Case report

## Abstract

**Background:**

Subhepatic appendicitis is an exceedingly rare presentation, accounting for 0.01% of acute appendicitis cases. It is of prime importance to be aware of variants and manage such challenging cases accordingly.

**Case presentation:**

We present a case of a middle-aged Saudi woman with subhepatic perforated appendicitis and peritonitis who underwent an exploratory laparotomy and appendectomy.

**Conclusions:**

The initial diagnosis and surgical management of such patients is challenging due to an atypical presentation. The surgical management of such patients is discussed with a brief review of the literature.

## Background

The appendix, a vestigial organ, is a small, tubelike structure that belongs to the midgut of the digestive tract system. The most common location of the appendix is retrocecal (74%), followed by the pelvic (21%) region. Other locations include subcecal (1.5%), preileal (1%), and postileal (0.5%) positions [1]. Acute appendicitis continues to be one of the most frequently encountered surgical emergencies in children and adults. The site of a normally placed appendix and its classical presentation of appendicitis are well documented in the literature. However, the deviations in the anatomical position of the appendix contribute to the difficulty in diagnosing appendicitis [2–8]. Subhepatic, left-sided, intraherniary, lateral pouch, mesocolic, and lumbar positions are rare positions of the appendix. It is of prime importance to be aware of variants and manage such challenging cases accordingly. Subhepatic appendicitis could mimic cholecystitis and liver abscess, resulting in delayed diagnosis and appendiceal rupture [1, 7].

We present a unique and challenging case of a middle-aged woman with subhepatic perforated appendicitis and peritonitis. The case is unique in its diagnosis and management, which are challenging. This case report will make readers aware of a rare presentation and its management. The surgical management of such patients is discussed along with a brief review of the literature.

## Case presentation

Our patient was a 41-year-old Saudi woman, a homemaker with no employment history and no known past medical history. She was not taking any home medications. She had no relevant or pertinent social, environmental, or family history and no prior smoking habit or alcohol consumption. She had a history of two normal vaginal deliveries followed by a cesarean section 1 year earlier, in August 2019. She presented to our hospital with abdominal pain of 3 days’ duration. The pain had started in the epigastric region, progressed in intensity over the 3 days, and became prominent in the right upper and lower quadrants. It was associated with one episode of nonbilious emesis and by mouth intolerance at home.

Upon presentation in the emergency department (ED), the patient was hypotensive with blood pressure of 90/40 mmHg, tachycardic with a heart rate of 112 beats/minute, and febrile to 38.2 °C, and she also showed signs of dehydration. She was conscious, alert, and oriented with a Glasgow Coma Scale score of 15, with unlabored breathing and normal vesicular breath sounds. Her abdominal examination showed a soft abdomen with tenderness to palpation in all the quadrants, prominently in the right upper and lower quadrants, and signs of peritonitis such as rebound tenderness and severe pain on percussion were present in the right abdomen. No musculoskeletal anomalies were observed, and distal pulses were present. The patient was given a 1-L bolus of Ringer’s lactate in the ED with a response of 100 mmHg systolic blood pressure.

**Fig. 1:**
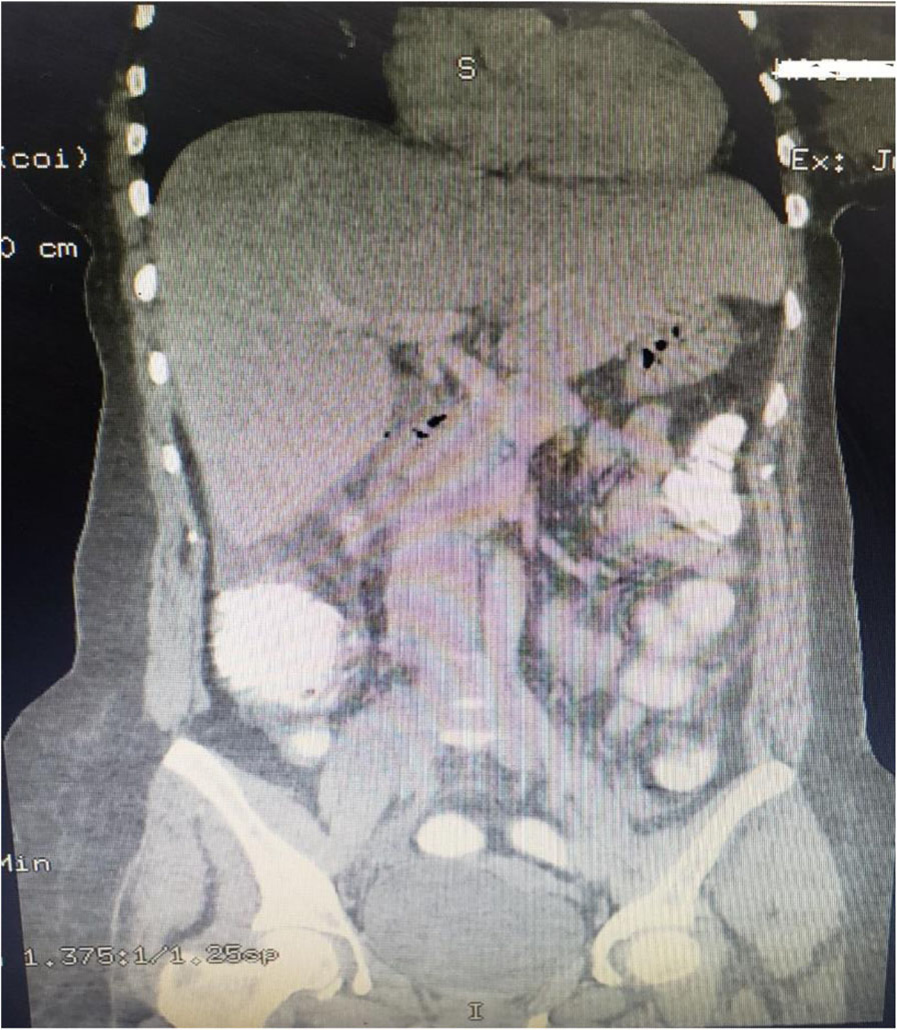
Computed tomographic images showing perforated subhepatic appendicitis with a fecalith

**Fig. 2:**
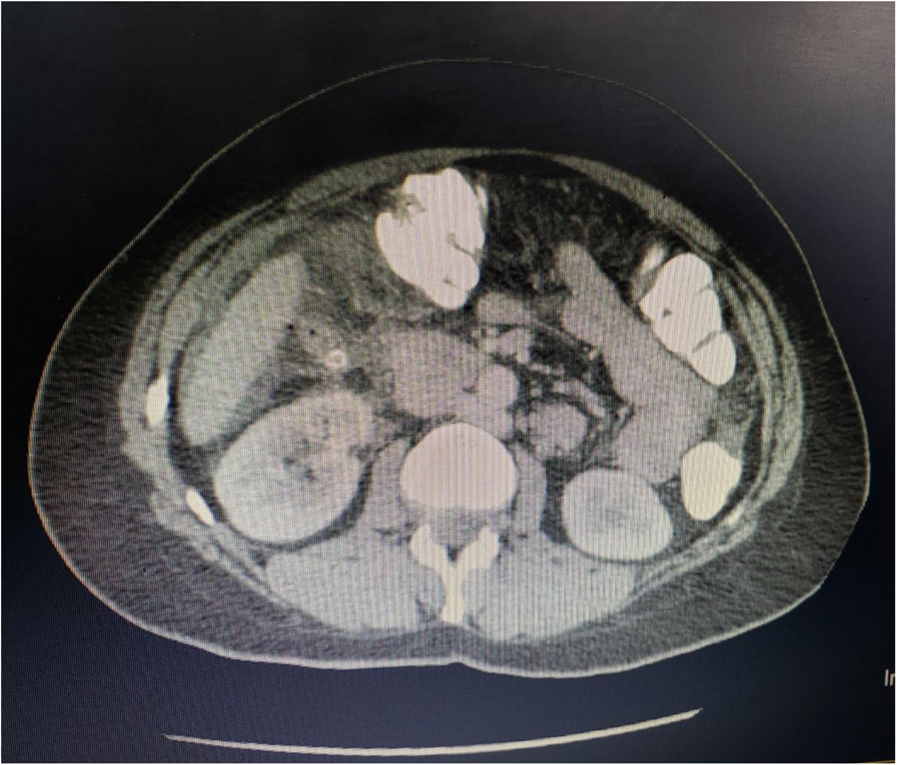
Computed tomographic images showing perforated subhepatic appendicitis with a fecalith

Laboratory tests were performed, which showed a white blood cell count of 11.8 × 10^9^/L, hemoglobin of 12.5 g/dl, platelet count of 320 × 10^9^/L, blood urea nitrogen 26 mg/dl, and creatinine of 0.75 mg/dl, as well as a normal liver function test result and normal coagulation profile. In addition, results of hepatitis B, hepatitis C, and human immunodeficiency virus testing were negative. Urine analysis showed no abnormal findings. An ultrasound of the patient’s abdomen showed subhepatic intraperitoneal fluid collection and inability to visualize the appendix. Axial computed tomography (CT) with by mouth and intravenous contrast showed subhepatic perforated appendicitis with subhepatic and pelvic collections (Figs. 1 and 2). The patient was started on intravenous ceftriaxone 1 g twice daily, intravenous metronidazole 500 mg thrice daily, and intravenous paracetamol 1 g thrice daily in the ED until discharge. On the basis of the CT findings and the clinical presentation, it was deemed necessary to proceed with an emergent laparotomy.

Under aseptic precautions and general anesthesia, the patient was placed in a supine position. A midline laparotomy incision was made. Upon entering the peritoneal cavity, a short ascending colon with a subhepatic perforated appendix acutely inflamed with a subhepatic collection was noticed. Localized peritonitis was present. A pyogenic membrane was noticed under the liver and between the liver and the diaphragm. A purulent collection was also noticed in the pouch of Douglas. Appendicectomy was performed. Complete hemostasis was achieved. Adequate peritoneal lavage was done with normal saline. After drainage of almost all the fluid, a right subhepatic drain and a left pelvic drain were placed. Abdominal wall closure of the rectus with a polydioxanone suture and skin staples was done. The patient was extubated in stable condition. No complications occurred.

The patient did well on postoperative day 1 (POD1) and tolerated her diet, and the drains were subsequently removed on POD2. The patient was discharged to home in a good condition and expressed gratitude. Postoperative follow-up at 2 weeks and at 6 months showed good healing and recovery of the patient.

## Discussion and conclusions

We present a unique and challenging case of a middle-aged woman with subhepatic perforated appendicitis and peritonitis. The case is unique in its diagnosis and management, which are challenging. This case report makes readers aware of a rare presentation and its management. The annual incidence rate of subhepatic appendicitis is approximately 0.09 per 100,000 population [2]. Incomplete rotation and fixation of the intestine due to a defect in fetal gut rotation results in a subhepatic cecum and appendix [9]. This is a very rare phenomenon. The earliest review of subhepatic cecum and appendix was documented in 1863, as reported in a review by King in 1955 [3]. Often mimicking hepatobiliary or gastric disease clinically, resulting in a delay in diagnosis of subhepatic appendicitis [1, 7]. This results complications such as sepsis, suppuration, and perforation [2]. Radiologic imaging thereby is of prime importance in identifying such an anomaly. Due to the availability and ease of performing ultrasound, ultrasound may be the preferred first-line screening modality. High suspicion and caution must be maintained in atypical presentations due to reports of subhepatic appendiceal disease misdiagnosed as liver abscess or cholecystitis [1, 2]. In our patient’s case, abdominal ultrasound showed subhepatic fluid collection and inability to visualize the appendix. CT of the abdomen and pelvis provides high sensitivity (100%), specificity (95%), and accuracy (98%) in identifying acute appendicitis [10]. In our patient, a CT scan delineated subhepatic perforated appendicitis with a subhepatic and pelvic collection. The appendix also contained a fecalith.

In a subhepatic appendix, a conventional Lanz incision in the right lower quadrant may not be suitable to remove the appendix. In our patient’s case, we performed a midline laparotomy due to the subhepatic location of the appendix and the possibility of retrocecal, dense adhesions or fibrosis and perforation, which would make a laparoscopic approach an unsafe option, in addition to the fact that open access would provide better tactile input and direct access to the appendix. Laparoscopy could also be an option in patients who are clinically stable and not peritonitic in a similar situation for its versatility and diagnostic and therapeutic ability [7]. If one were to proceed laparoscopically, steps that would be beneficial include using an angled laparoscope for better viewing, initial mobilization of the cecum, using an extra port for better access, and twisting of the appendix, making dissection easier.

In conclusion, subhepatic appendicitis is a unique and rare presentation, making its diagnosis and management challenging. Surgeons must be cognizant of this atypical presentation and how patients can present late due to considering other possible nonsurgical causes such as gastritis or biliary colic. Surgeons must also be aware of the various discussed surgical modalities.

## Data Availability

Not applicable.

## Abbreviations

CT: Computed tomography
ED: Emergency department
POD: Postoperative day
